# Phytochemical Characterization of *Astragalus boeticus* L. Extracts, Diuretic Activity Assessment, and Oral Toxicity Prediction of *Trans*-Resveratrol

**DOI:** 10.3390/ph18121893

**Published:** 2025-12-15

**Authors:** Ahmed Elfallaki Elidrissi, Najoua Soulo, Amal Elrherabi, Tarik Chelouati, Otmane Zouirech, Abdelkrim Agour, Karima El-Yagoubi, Widad Tbatou, Fahd A. Nasr, Mohammed Al-zharani, Ashraf Ahmed Qurtam, Elhoussine Derwich

**Affiliations:** 1 Laboratory of Biotechnology, Conservation, and Valorization of Bioresources (BCVB), Research Unit: Api-Phytotherapy, Physiology, Environment, and Health, Department of Biology, Faculty of Sciences Dhar Mahraz, Sidi Mohamed Ben Abdellah University, Fez 30000, Morocco; ahmed.elfallakielidrissi@usmba.ac.ma (A.E.E.); najoua.soulo@usmba.ac.ma (N.S.); tarik.chelouati@usmba.ac.ma (T.C.); otmane.zwirech@usmba.ac.ma (O.Z.); abdelkrimagour1@gmail.com (A.A.); karima.elyagoubi@usmba.ac.ma (K.E.-Y.); widad.tbatou@usmba.ac.ma (W.T.); elhoussinederwich@yahoo.fr (E.D.); 2 Laboratory of Bioresources, Biotechnology, Ethnopharmacology and Health, Faculty of Sciences, University Mohammed 1st, Bd. Med VI B.P. 717, Oujda 60000, Morocco; 3 Biology Department, College of Science, Imam Mohammad Ibn Saud Islamic University (IMSIU), Riyadh 11623, Saudi Arabia; mmyalzahrani@imamu.edu.sa (M.A.-z.); aaqurtam@imamu.edu.sa (A.A.Q.)

**Keywords:** *Astragalus boeticus*, diuretics, ProTox-II, *trans*-resveratrol, LC-MSMS, plant bioactives

## Abstract

**Background/Objectives:** Plant-derived diuretics are attracting increasing interest due to their promising efficacy and improved safety profile compared with synthetic drugs. This study aimed to characterize the phytochemical composition of *Astragalus boeticus* (*A. boeticus*) extracts, evaluate their diuretic activity, and assess the oral safety of their main phenolic compound. **Methods:** Aqueous (AQE) and hydroethanolic (EtOHE) extracts were analyzed using LC–MS/MS, while in silico toxicity prediction of *trans*-resveratrol was performed using ProTox-II and ADMETlab 2.0. Diuretic activity was evaluated in male Wistar rats (n = 24) divided into four groups: control (distilled water, 10 mL/kg), furosemide (10 mg/kg), AQE (300 mg/kg), and EtOHE (300 mg/kg). Urine and plasma samples were collected after 15 days to determine electrolyte concentrations, creatinine level, creatinine clearance, and hepatic enzyme profile. **Results:** LC–MS/MS profiling identified fourteen phenolic compounds, with *trans*-resveratrol (270 µg/g in AQE) being the most abundant, followed by cyanidin-3-O-glucoside and gentisic acid. In silico assessments revealed no hepatotoxic, mutagenic, or neurotoxic effects of *trans*-resveratrol. Both extracts significantly enhanced urinary output, chloride excretion, and creatinine clearance, while maintaining stable renal and hepatic biochemical parameters, indicating potent diuretic activity without toxicity. **Conclusions:** *A. boeticus* extracts demonstrate strong diuretic potential associated with a favorable safety profile, likely linked to their phenolic composition dominated by *trans*-resveratrol. These findings support the use of *A. boeticus* as a natural and safe diuretic source. Further investigation is recommended to elucidate its pharmacological mechanisms and therapeutic relevance.

## 1. Introduction

Synthetic diuretics, such as furosemide and hydrochlorothiazide, are commonly prescribed to manage conditions like hypertension and edema. However, their use is often associated with adverse effects, including electrolyte imbalances, dehydration, and hypotension [1,2,3]. These side effects highlight the need for alternative therapeutic options. Botanical diuretics, derived from plant sources, have gained attention as potential substitutes due to their natural origin and perceived safety [4,5,6]. For instance, the ethanolic extract of *Annona squamosa* leaves demonstrated significant diuretic and antiurolithiatic activities in experimental models [7]. Numerous plant species have demonstrated diuretic properties with various mechanisms of action [8,9]. Particularly, members of the Fabaceae family, such as *Mucuna pruriens* and *Senna septemtrionalis*, have shown significant diuretic effects comparable to synthetic diuretics [5,6]. These plants contain bioactive compounds like flavonoids and alkaloids, which may contribute to their diuretic activity [4,7]. Despite the promising diuretic effects of plant extracts, it is crucial to evaluate their safety profiles [8,9,10]. Toxicity assessments, including acute and subchronic studies, are essential to ensure the safe use of these botanicals. For instance, the evaluation of *Boscia coriacea* and *Uvaria leptocladon* leaf extracts revealed no significant toxicity, supporting their safe use in traditional medicine [9]. Additionally, acute and subacute toxicity studies of *Justicia schimperiana* leaf extracts indicated no mortality or significant weight loss, confirming their safety [8,11].

Among the phenolic compounds found in plant extracts, *trans*-resveratrol is known for several significant therapeutic properties. Studies indicate that *trans*-resveratrol exerts notable neuroprotective actions by enhancing mitochondrial efficiency and limiting pathological protein aggregation [12]. Its anticancer potential is supported by evidence showing its ability to regulate apoptosis, inhibit abnormal cell proliferation, and suppress angiogenesis [13]. Moreover, improvements in endothelial function reinforce its recognized cardioprotective effects [14]. Earlier investigations confirmed its anti-inflammatory capacity, particularly through inhibition of NF-κB–related pathways [15]. Foundational research also demonstrated that *trans*-resveratrol is a potent antioxidant capable of reducing oxidative stress associated with chronic diseases [16].

Among the various species within the *Astragalus* genus, *A. boeticus* remains relatively underexplored. This study aims to perform a comprehensive phytochemical characterization of aqueous and ethanolic extracts of *A. boeticus* using Liquid Chromatography–Tandem Mass Spectrometry (LC-MS/MS), evaluate their diuretic activity, and predict, via in silico approaches, the toxicity of the major phenolic constituent using ProTox-II and ADMETlab 2.0, thereby providing a scientific foundation for their potential therapeutic applications.

## 2. Results

### 2.1. Phytochemical Identification of the Extracts

The extracts (AQE and EtOHE) analyzed by LC-MSMS included numerous phenolic compounds found in this medicinal plant. We identified and reported fourteen compounds, including four hydroxycinnamic acids, three anthocyanidins, a stilbene, a flavonol, and five benzoic acids (Table 1). The compounds were quantified using calibration curves for phenolic compounds with very similar structures.

Myricetin (5 µg/g), Cyanidin-3-O-glucoside (90 µg/g), Petunidin-3-O-glucoside (1 µg/g), and Malvidin-3-O-glucoside (43 µg/g) are among the flavonoids present in AQE, along with some phenolic acids, including Protocatechuic acid (40 µg/g), Vanillic acid (0.075 µg/g), Gallic acid (22 µg/g), Gentisic acid (97 µg/g), p-Coumaric acid (75 µg/g), Salicylic acid (7 µg/g), Caffeic acid (1 µg/g), Ferulic acid (76 µg/g), Sinapic acid (97 µg/g), and the stilbene Trans-resveratrol. Notably, cyanidin-3-O-glucoside (90 µg/g) is the main flavonoid in the extract. Some phytochemical compounds are not detectable, such as p-hydroxybenzoic acid, flavan-3-ols, kaempferol, quercetin, and isorhamnetin. These may be phenolic substances not required for our analysis. Trans-resveratrol (170 µg/g), cyanidin-3-O-glucoside (90 µg/g), gentisic acid (89 µg/g), vanillic acid (81 µg/g), and p-coumaric acid (67 µg/g) are the primary constituents of EtOHE.

### 2.2. Predictive Toxicological Characterization of Trans-Resveratrol from A. boeticus

**Figure 1:**
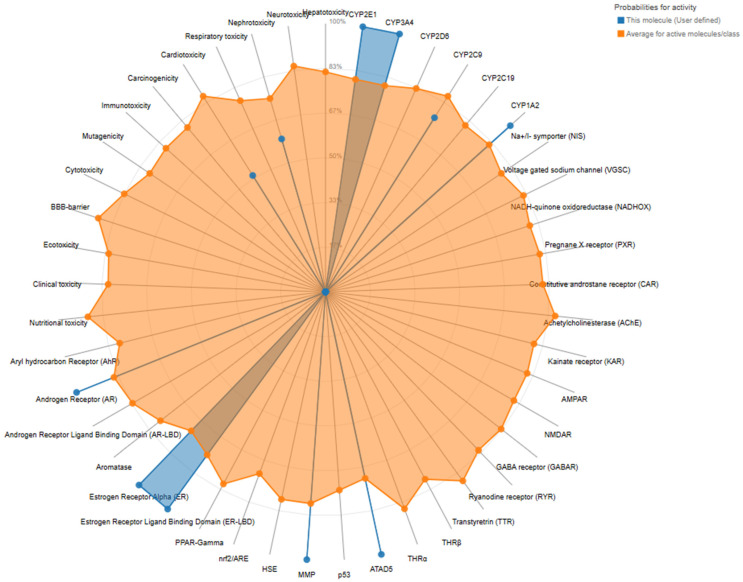
Toxicity radar chart is intended to quickly illustrate the confidence of positive toxicity results compared to the average of its class.

The in silico assessment of oral toxicity for *trans*-resveratrol, the major phenolic constituent of *A. boeticus* extracts, indicated an overall favorable safety profile across multiple computational endpoints. The predicted toxicological responses and molecular target interactions, summarized in Table 2 and Table 3**,** provide insights into the compound’s potential effects on diverse biological systems and its affinities toward specific molecular receptors. Additionally, Figure 1 offers a graphical overview of the confidence levels associated with positive toxicity predictions, facilitating a comprehensive visualization of their relative significance. According to the predictive models (ProTox-II and ADMETlab 2.0), trans-resveratrol demonstrated low probabilities for most organ-related toxicities, being classified as inactive for hepatotoxicity (0.74), neurotoxicity (0.77), and respiratory toxicity (0.57). Slight activity was observed for nephrotoxicity (0.59) and cardiotoxicity (0.51), although these probabilities remain below the threshold of toxicological concern, suggesting limited potential for adverse renal or cardiac effects under physiological exposure conditions.

Regarding the general toxicity endpoints, *trans-resveratrol* was predicted to be inactive for carcinogenicity (0.71), immunotoxicity (0.86), mutagenicity (0.92), cytotoxicity (0.98), and ecotoxicity (0.55). Furthermore, predictions for blood–brain barrier permeability, clinical toxicity, and nutritional toxicity were also negative. These results indicate that the compound possesses a negligible risk of genotoxic, immunosuppressive, or cytotoxic effects, corroborating its established safety profile as a dietary polyphenol.

In the context of nuclear receptor signaling pathways, *trans-*resveratrol exhibited strong predicted activation of the androgen receptor (AR), estrogen receptor alpha (ER), and estrogen receptor ligand-binding domain (ER-LBD), each with the highest probability (1.0). Such results are consistent with previous reports describing its phytoestrogenic potential and modulatory influence on steroid hormone signaling. Additionally, the compound showed activity toward the mitochondrial membrane potential (MMP; 1.0) and the DNA repair-related protein ATAD5 (1.0), suggesting potential roles in mitochondrial homeostasis and genomic stability. These interactions may contribute to its known antioxidant and cytoprotective activities.

Metabolic pathway predictions indicated that *trans-*resveratrol is primarily biotransformed by cytochrome P450 isoenzymes CYP1A2 (0.92), CYP2C9 (0.76), and CYP3A4 (1.0), while other isoforms such as CYP2C19, CYP2D6, and CYP2E1 remained inactive. These data suggest that the compound may undergo oxidative metabolism mainly through phase I reactions, with potential implications for drug–drug interactions involving these CYP isoforms.

Taken together, these in silico findings reinforce the oral safety and pharmacological potential of *trans-*resveratrol, supporting its continued consideration as a bioactive compound of therapeutic interest. The moderate predictions for nephrotoxicity and cardiotoxicity are unlikely to translate into significant adverse effects at physiological concentrations but warrant further validation through in vivo and clinical investigations. The predicted interactions with nuclear receptors and mitochondrial pathways highlight the compound’s multifaceted mechanism of action, which may underpin the diuretic and antioxidant effects observed in *A. boeticus* extracts.

### 2.3. Subchronic Diuretic Treatment

#### 2.3.1. Impact of AQE and EtOHE on Urinary Electrolyte Excretion

**Figure 2:**
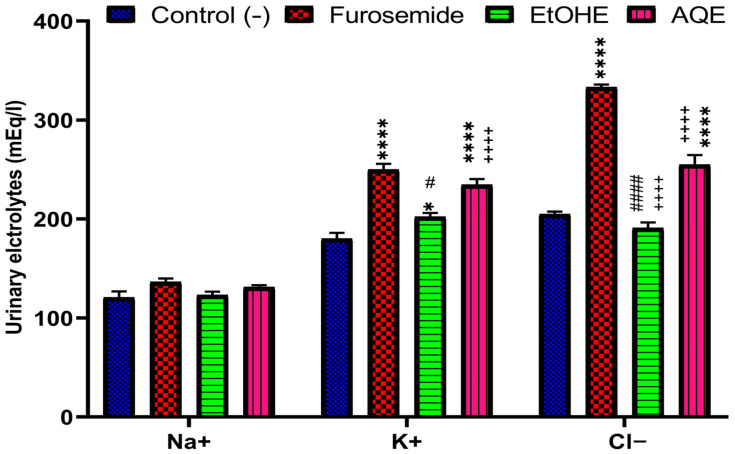
Effects of AQE, EtOHE, Furosemide, and distilled water on Na+, K+, and Cl^−^ excretion at day 15. * Comparison between all groups and the control group. + Comparison between the extract groups and the Furosemide group. **#** Comparison between AQE and EtOHE * *p* < 0.05, **** *p* < 0.0001; ++++ *p* < 0.0001; **#** *p* < 0.1, **####** *p* < 0.0001.

The following data summarizes the impacts of AQE, EtOHE, and furosemide on the urinary clearance of chloride, sodium, and potassium (Figure 2). Furosemide, along with the 20 mg/kg dosage of AQE and EtOHE, resulted in a significant increase (*p* < 0.0001) in urinary clearance of Na+ and Cl− compared to the baseline.

However, potassium excretion levels were 202.33 ± 3.84 mEq/L for EtOHE and 234.67 ± 5.70 mEq/L for AQE. For furosemide, the increase recorded was 250 ± 5.77 mEq/L. Furthermore, the oral administration of *A. boeticus* extracts over two weeks led to a significant rise in chloruresis compared to the group that drank distilled water. For EtOHE, this increase was 191 ± 5.57 mEq/L, while for AQE, it was 255.13 ± 9.58 mEq/L. Furosemide treatment also caused higher chloruresis (333.33 ± 2.60 mEq/L) compared to the normal group (205 ± 2.65 mEq/L). In contrast, although slight variations were observed, urinary sodium levels did not show any statistically significant changes after 15 days of gavage with AQE, furosemide, or EtOHE (Figure 2).

The saluretic index and Na^+^/K^+^ ratio for each of the four groups (Control, Furosemide, AQE, and EtOHE) are shown in Table 4. The sodium, potassium, and chloride excretion values of the reference group, also known as the Control group, are all set to 1. Compared to the Control, the Furosemide group has higher values for sodium (1.12), potassium (1.38), and chloride (1.6), along with a Na^+^/K^+^ ratio of 0.54. The Na^+^/K^+^ ratio of 0.55 indicates that the AQE group has the highest levels of sodium (1.08), potassium (1.3), and chloride (1.24) among all treatments. The EtOHE group showed significant increases in potassium (1.12) and sodium (1.02), with a Na^+^/K^+^ ratio of 0.6.

#### 2.3.2. Impact of AQE and EtOHE on Urinary Urea and Creatinine

**Figure 3:**
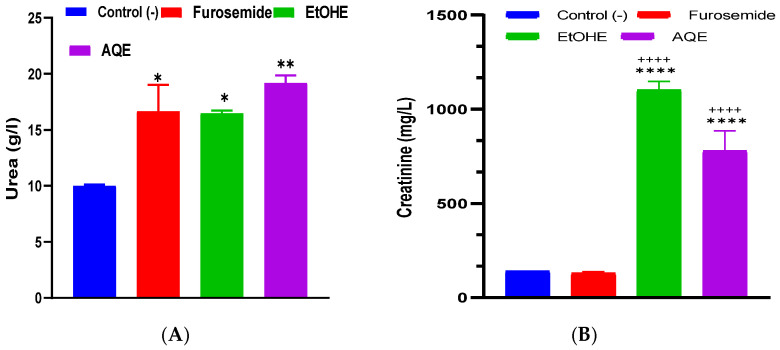
Impact of AQE, EtOHE, Furosemide, and distilled water on the excretion of urea (**A**) and creatinine (**B**). * Comparison among all groups and the control group; + comparison between the extract groups and the Furosemide group. * *p* < 0.05, ** *p* < 0.01, **** *p* < 0.0001; ++++ *p* < 0.0001.

Figure 3 illustrates the effects of 15 days of treatment with AQE, EtOHE, and furosemide on urinary urea and creatinine levels in rats. For urea (Figure 3A), the control group exhibited the lowest concentration, whereas furosemide, EtOHE, and AQE significantly increased urinary urea excretion, with AQE producing the strongest elevation (* *p* < 0.05 to * *p <* 0.01). For creatinine (Figure 3B), both EtOHE and AQE induced a marked rise in urinary creatinine levels compared to the control and furosemide groups, with EtOHE showing the highest increase (**** *p* < 0.0001). Furosemide produced only a minimal, non-significant effect.

These results indicate that both AQE and EtOHE improve renal excretion of nitrogenous waste, as shown by increased urinary urea and creatinine levels. The greater increases seen with EtOHE and AQE may suggest enhanced glomerular filtration or stimulation of renal elimination processes. Importantly, the changes are consistent with a diuretic effect rather than renal damage.

#### 2.3.3. Impact of AQE and EtOHE on Plasma Electrolyte Level

**Figure 4:**
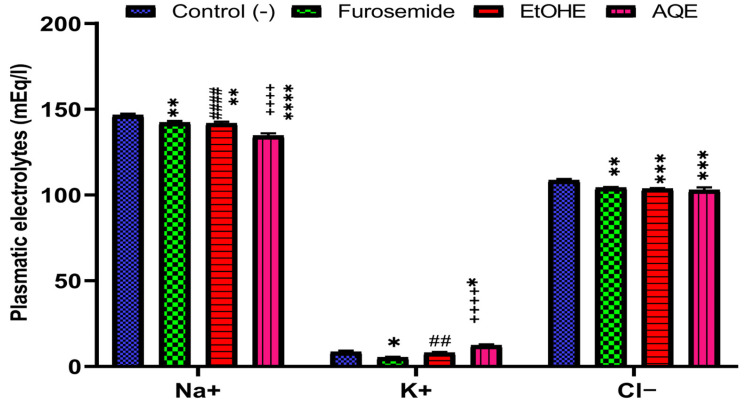
Effects of AQE and EtOHE, and Furosemide on serum Na+, K+, and Cl^−^ at day 15 * Comparison between all groups and the control group. + Comparison between the extract groups and the Furosemide group. **#** Comparison between AQE and EtOHE; * *p* < 0.05, ** *p* < 0.01, *** *p* < 0.001, **** *p* < 0.0001; ++++ *p* < 0.0001; **##** *p* < 0.01, **####** *p* < 0.0001.

Unlike the control group, administering AQE and EtOHE for 15 days caused changes in plasma chloride, sodium, and potassium levels. However, furosemide and both extracts led to a significant decrease in plasma sodium and potassium levels (*p* < 0.05) and an increase in plasma potassium levels (*p* < 0.05) (Figure 4).

#### 2.3.4. Impact on Plasma Urea and Creatinine Levels

**Figure 5:**
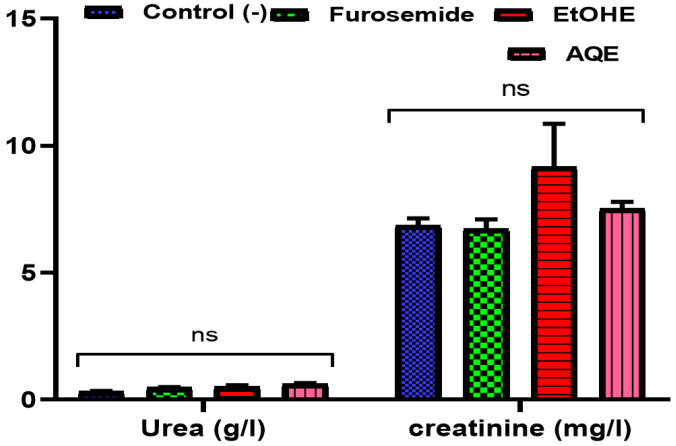
Impact of aqueous and hydroethanolic extracts administered orally of *A. boeticus* and furosemide on renal function. ns: non-significative.

Serum urea and creatinine did not significantly change after fifteen days of treatment with AQE and EtOHE of both *A. boeticus* and furosemide (Figure 5).

#### 2.3.5. Impact of AQE and EtOHE on Creatinine Clearance

**Figure 6:**
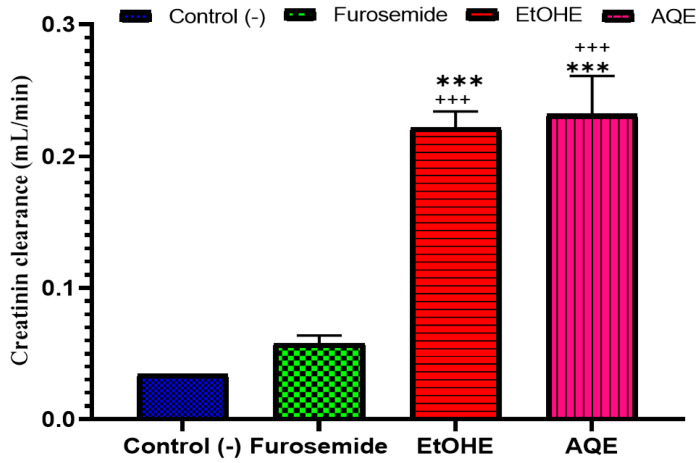
Plasma creatinine clearance (mL/min) in rats as a result of oral administration of AQE, EtOHE, furosemide, and pure water (mean ± SEM). * Comparison between all groups and the control group. + Comparison between the extract groups and the Furosemide group. *** *p* < 0.001; +++ *p* < 0.001.

Extracts of *A. boeticus* caused a significant increase (*p* < 0.001) in creatinine clearance on the final day of treatment, compared to the control group. More importantly, this increase was seen in the groups that received aqueous and hydroethanolic extracts, compared to the furosemide group (Figure 6).

#### 2.3.6. Impact of AQE and EtOHE on Hepatic Biochemical Parameters

**Figure 7:**
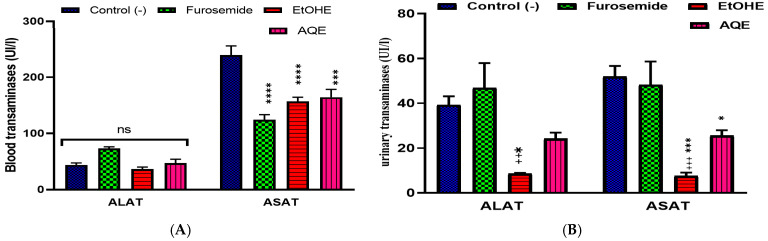
Comparative Profile of Serum (**A**) and Urinary (**B**) Transaminases in Control, Furosemide, and Extract-Treated Groups; * Comparison between all groups and the control group. + Comparison between the extract groups and the Furosemide group. * *p* < 0.05, *** *p* < 0.001, **** *p* < 0.0001; +++ *p* < 0.001, ++ *p* < 0.01.

Figure 7A illustrates the effects of the treatments on serum transaminases (ALAT and ASAT). ALAT levels remained relatively stable across all groups, with only minor fluctuations, while ASAT activity was significantly higher in the control group and considerably lower in the furosemide, AQE, and EtOHE groups. Figure 7B shows the profile of urinary transaminases. Both ALAT and ASAT excretions were markedly reduced in the EtOHE group compared to the control, whereas the AQE group exhibited intermediate values, and furosemide resulted in higher urinary levels. Statistical markers indicate significant differences between treated and control animals.

## 3. Discussion

The purpose of this study was to examine the phytochemical constituents and assess the diuretic effects of oral AQE and EtOHE administration in normal Wistar rats. Due to its potential therapeutic benefits, *A. boeticus*, a common plant used in traditional medicine worldwide, has attracted considerable attention. Phenolic compounds are very prevalent in the *Astragalus* species [17]. They have been the subject of much research and show considerable antioxidant activity, which prevents oxidative stress [18], and are often associated with renal and cardiovascular problems [19]. Stress brought on by oxygen enhances renal function and encourages sodium reabsorption, which lowers diuresis. Polyphenols improve renal vasodilation, shield renal tissues, and encourage the outflow of water and electrolytes by preventing reactive oxygen species (ROS) [20]. This antioxidative action may therefore account for a portion of the diuretic impact seen in our extracts, which are high in phenolic chemicals. Polyphenols may provide a basis for the development of new medications. Furthermore, polyphenolic bioactive substances, which are prevalent in plant extracts and include flavonoids and phenolic acids, are the most important constituents of goods obtained from plants. However, the chemical makeup of plants is influenced by climatic elements such as soil type, weather, and botanical and geographic origins [21]. Furthermore, the results of this study’s phytochemical analysis showed that AQE and EtOHE included fourteen polyphenolic components, such as gallic acid, p-coumaric acid, Myricetin, cyanidin-3-O-glucoside, and gentisic acid. These outcomes concur with those attained by Aydemir et al. (2024) [22], where LC–ESI–MS/MS is used to quantify five substances (Hesperidin, hyperoside, p-hydroxybenzoic acid, protocatechuic acid, and p-coumaric acid) in *Astragalus gymnolobus* species collected from Turkey. Within the same framework, an additional study carried out by Cengiz Sarikurkcu and Gokhan Zengin (2020) [23] identified hyperoside, apigenin, p-coumaric, and ferulic acids within the phenolic composition of the methanolic extract of *Astragalus macrocephalus* subsp. *finitimus* collected from Sucati village, Gurun, Sivas-Turkey.

Being the first to investigate the diuretic effects of *A. boeticus*’s AQE and EtOHE in Morocco, the current study is special. Diuretic drugs can help with several ailments, such as liver cirrhosis, hypertension, and hypercalciuria. It is crucial to show that they efficiently control electrolytes and water balance because they are used therapeutically to treat edema [3]. The diuretic effects of the extracts were tested in this investigation using normal rats. The results showed that when taken daily for 15 days, AQE and EtOHE had a beneficial effect on diuretic activity. Furosemide was used as our reference drug to assess the diuretic effect of our sample. Diuretics increase the output of urine and aid in lowering fluid accumulation linked to diseases such as high blood pressure, heart disease, and renal illness [24,25]. Clinical diuretics are crucial for controlling the volume of extracellular fluid and greatly increasing the excretion of Na+. Furosemide blocks the Na+ -K+ -2 Cl- symporter, which interferes with the absorption of Na+ [26]. Both *A. boeticus* extracts employed in this study (AQE and EtOHE) showed a strong ability to improve urine flow rate, with efficacy on par with that of the common medication, furosemide. With just a slight effect on plasma electrolyte levels, a notable rise in potassium and chloride excretion was noted in the urine. This suggests that *Astragalus boeticus* might promote kaliuresis and chlorurie by acting as a loop diuretic.

Since the excretion of electrolytes (Na^+^, K^+^, and Cl^−^) in the AQE group is comparable to that in the EtOHE group, the saluretic index shows a more noticeable diuretic effect. Additionally, the Na^+^/K^+^ ratio in the EtOHE-treated group (0.60) is marginally higher than that of the furosemide group (0.54), indicating a comparatively more favorable potassium excretion, even if it is lower than that of the control group (0.67). This suggests that, in contrast to furosemide, extracts have a favorable diuretic effect by encouraging potassium excretion more than sodium [27]. Additionally, after taking AQE and EtOHE orally, creatinine clearance considerably increased. These outcomes are similar to those of pollen extract [28], *Solanum elaeagnifolium* [29], and *Pinus pinaster* Bark extract [30].

Higher electrolyte elimination and greater water excretion (urine production) are the two main indicators of diuresis. By inhibiting the Na/K/2 Cl symporter in the thick ascending limb of the loop of Henle, furosemide increases urine flow and sodium loss [31,32]. Although the exact mode of action of AQE and EtOHE investigated in this study is unknown, they appear to have a diuretic impact because they made it easier for potassium and chloride to be eliminated from the urine. However, it cannot be categorized as a loop diuretic like furosemide because no significant natriuretic response was shown.

It is still unknown which active ingredient gives *A. boeticus*’s aqueous and hydroethanolic extracts their diuretic effects. The presence of flavonoids, stilbenes, and phenolic acids, which have antioxidant and anti-inflammatory properties [33], can help improve blood vessel endothelial function [34], which, according to qualitative phytochemical screening of AQE and EtOHE, increases blood flow to the kidneys, potentially influencing renal function and increasing urine production. These drugs have been studied for their potential effects on a variety of illnesses, such as urinary tract infections, bladder dysfunction, and benign prostatic hyperplasia (BPH) [35].

Furthermore, some studies have shown that polyphenols have a diuretic impact [36,37]. According to Zeng et al. (2019) [37], some phenolic compounds, especially flavonoids, can increase kaliuresis and natriuresis. It has been demonstrated that flavonoids increase sodium excretion and diuresis by binding to the adenosine A1 receptor [36]. Accordingly, HPLC phytochemical analyses have demonstrated that this plant’s extracts contain a significant number of flavonoids. Therefore, the plant’s diuretic activity can be due to its abundance of flavonoids. However, recent research has shown that plant diuretic activity can also be caused by other secondary metabolites, especially saponins, which can change membrane permeability and encourage renal salt excretion [38,39].

Trans-resveratrol, gentisic acid, vanillic acid, gallic acid, cyanidin 3-O-glucoside, and p-coumaric acid were the most predominant polyphenolic components identified in our extracts. Growing research has demonstrated that in normal rats, gallic acid significantly increased urine output and electrolyte excretion, thereby demonstrating a potent diuretic effect [40]. Furthermore, gentisic acid significantly enhanced renal function by promoting natriuresis and diuresis, thereby contributing to improved renal clearance in experimental models [41]. Moreover, Vanillic acid exerts cardioprotective effects primarily through its antioxidant and anti-inflammatory properties, by inhibiting pro-inflammatory signaling pathways, reducing oxidative stress, and modulating endogenous antioxidant defenses [42]. Cyanidin-3-O-glucoside (C3G) exerts cardioprotective effects in hypertensive rats by preventing cardiomyocyte death and hypertrophy and reducing cardiac fibroblast activation, although it does not lower blood pressure [43]. Together, these compounds exhibit diuretic potential, promoting sodium excretion and reducing arterial pressure, thereby enhancing their cardioprotective effects [44].

The reduction in serum and urinary ASAT and ALAT activities in the extract-treated groups suggests a possible **hepatoprotective and renoprotective effect** of *A. boeticus*. Similar findings have been reported for other polyphenol-rich extracts, where attenuation of transaminase leakage reflected preserved tissue integrity and reduced oxidative damage [45,46]. Urinary transaminases, although less frequently assessed, have been proposed as early markers of tubular injury [47]. The significant reduction observed, particularly with the EtOHE extract, therefore supports the hypothesis that phytochemicals in *A. boeticus* may counteract the nephrotoxic side effects often associated with diuretics such as furosemide.

The results demonstrated that rats given aqueous and hydroethanolic extracts of *A. boeticus* (300 mg/kg body weight) subchronically exhibited a notable diuresis. Our investigation found no mortality for the dosage used. Thus far, however, thorough toxicological studies in mice and rats have not been performed. Evaluation of any side effects, such as modifications to neurological, metabolic, and hormonal processes not covered in this study, is crucial before suggesting this plant for clinical use. AQE and EtOHE’s precise areas of action, as well as their molecular and cellular mechanisms of action, require more investigation.

## 4. Materials and Methods

### 4.1. Plant Material

Samples of *A. boeticus* were collected during the period (March-April and May 2023) in the Ben Slimane forest (Casablanca region, Morocco) and identified at the Scientific Institute of Rabat, Morocco (Prof. Hamid Khamar). The plant was registered in the Herbarium index of the same institute under the number RAB 114143. The leaves were washed and dried away from sunlight in a well-ventilated room within the FSDMF laboratory at room temperature for four weeks, then pulverised using an electric grinder to produce a powder, which was used to prepare the extracts.

### 4.2. Extract Preparation

The extracts (aqueous, 70% ethanol) were obtained by macerating 100 g of powdered leaves in 1 L of the respective solvent for 48 h under magnetic stirring. After filtration (Whatman paper), the filtrates were evaporated at 45 °C, weighed to determine extraction yields in relation to dry plant material, and stored at 4 °C for subsequent analyses.

### 4.3. Analysis by LC-MS/MS of A. boeticus Extracts

The phytochemical profiling of *A. baeticus* L. leaf extracts was conducted using high-performance liquid chromatography coupled with tandem mass spectrometry (LC–MS/MS) on a triple quadrupole mass spectrometer (TSQ Fortis, Thermo Scientific, Waltham, MA, USA). Prior to analysis, the extracts were dissolved in methanol, filtered through a 0.22 µm nylon syringe filter, and transferred into autosampler vials. All solvents and reagents utilized during extraction, purification, and analysis were of analytical grade. Chromatographic separation was achieved on a Phenomenex C18 column (100 × 2.1 mm, 2.2 µm) maintained at 35 °C. The mobile phase system consisted of solvent A (0.1% formic acid and 10% methanol in water) and solvent B (0.1% formic acid in acetonitrile). The gradient elution program was as follows: 0–2 min, 95% A; 2–7 min, 95–85% A; 7–15 min, 85–50% A; 15–18 min, 50–20% A; 18–19 min, held at 20% A; and 19–21 min, re-equilibration to 95% A. The injection volume was 2 µL, with a constant flow rate of 0.3 mL/min. Mass spectrometric detection was performed in positive electrospray ionization (ESI^+^) mode, using a capillary voltage of 3500 V, with a mass scan range of *m*/*z* 50–1200 and a scan rate of 2 Hz. Compound identification was carried out in multiple reaction monitoring (MRM) mode, focusing on polyphenolic and related phenolic constituents by monitoring precursor-to-product ion transitions, ensuring high selectivity and sensitivity. Data acquisition and processing were accomplished using Thermo TraceFinder software.

### 4.4. In Silico Toxicological Profiling of Trans-Resveratrol from A. boeticus

#### 4.4.1. Phytochemical Target Compound

The major phenolic constituent identified in *A. boeticus* extracts was trans-resveratrol, a stilbene derivative widely recognized for its broad pharmacological potential. This compound was selected for in silico toxicity evaluation due to its quantitative predominance in the extract and its biological relevance as a natural antioxidant.

The molecular descriptors of trans-resveratrol were analyzed to determine its physicochemical and pharmacokinetic suitability as a bioactive molecule. The main molecular characteristics are summarized in Table 5.

#### 4.4.2. Toxicological Evaluation

The oral toxicity and safety profile of *trans*-resveratrol were investigated through in silico predictive approaches employing advanced cheminformatic and machine-learning methodologies. Toxicological simulations were conducted using two complementary computational platforms: ProTox-II [48], designed to predict organ-specific toxicity, general toxicological endpoints, nuclear receptor modulation, and stress response pathways; and ADMETlab 2.0 [49], which estimates pharmacokinetic behaviors including absorption, distribution, metabolism, excretion, and potential interactions with cytochrome P450 isoenzymes. The canonical SMILES structure of *trans*-resveratrol was submitted as input for both predictive systems, generating qualitative outcomes (“Active” or “Inactive”) accompanied by probabilistic confidence scores ranging from 0 to 1. All analyses were performed under default computational parameters, and the resulting toxicological profiles were systematically categorized into five principal domains: organ toxicity, general toxicity endpoints, nuclear receptor signaling, stress response pathways, and CYP-mediated metabolic interactions.

### 4.5. Evaluation of the Diuretic Effect of A. boeticus Extracts

#### 4.5.1. Animals

Sixteen male Wistar rats (190 ± 11 g), were used to achieve this experiment. The breeding of these animals is done in the animal house of Sidi Mohamed Ben Abdallah University. Indeed, they were housed in cages where food and tap water were freely available, under ambient temperature conditions (25 °C), with a natural cycle of 12 h of light followed by 12 h of darkness. The research was conducted by ethical guidelines for the use and care of laboratory animals, and approval from the Ethical Committee, Faculty of Sciences, Fez, Morocco, was obtained (USMBA-SNAMOPEQ 2021). All efforts were made to minimize animal suffering and the number of animals used.

#### 4.5.2. Experimental Design

The diuretic activity was evaluated following the protocol described in the study of El-Guendouz et al. (2017) [50]. Before beginning the experiment, each rat was put into a metabolic cage for 48 h. Four groups (n = 4) were employed and subjected to a 12 h food deprivation period before testing, with access to water.

The control group (Group 1) received 10 mL/kg body weight of distilled water orally via gavage. Group 2 was administered with 10 mg/kg body weight of furosemide orally via gavage. Group 3 received 300 mg/kg body weight of EQE, while Group 4 received a higher dose of 300 mg/kg body weight of EtOHE.

On treatment days 0 and 15, urinary samples were collected using graduated cylinders to quantify water excretion. Subsequently, the samples underwent filtration, centrifugation, and preservation for the analysis of creatinine, sodium, and potassium, chlore, ALAT and ASAT levels.

The animals were anesthetized using diethyl ether, and blood samples were collected from the retro-orbital plexus using heparinized capillary tubes at the end of the treatment [28]. After that, the plasma was separated by centrifugation at 10,000× *g* for 10 min, and it was kept at −20 °C so that creatinine, sodium, and potassium, chlore, ALAT and ASAT could be measured [51]. Statistical analyses were performed using GraphPad Prism^®^ 8, applying one-way ANOVA followed by appropriate post hoc tests to determine significant differences between groups.

## 5. Conclusions

The findings of this study demonstrate that *A. boeticus* aqueous and hydroethanolic extracts exhibit notable diuretic activity, as evidenced by increased urinary output, chloride excretion, and creatinine clearance, without observable alterations in basic hepatic or renal parameters. LC-MS/MS analysis identified trans-resveratrol as the major phenolic constituent; however, the safety insights derived from in silico predictions for this single compound and the general long-term observations remain preliminary. Consequently, while the extracts show promising diuretic potential, the current data do not allow for definitive conclusions regarding their safety profile. Future investigations are essential, particularly comprehensive in vivo toxicity studies, detailed dose–response analyses, pharmacodynamic validations, mechanistic explorations, and ultimately human clinical evaluations, to better define the therapeutic relevance and clinical applicability of *A. boeticus* extracts.

## Figures and Tables

**Table 1:** Polyphenol compounds identified using LC-MSMS in *A. boeticus* aqueous and hydroethanolic extracts (µg/g).

Polyphenols	AqE (µg/g)	EtOHE (µg/g)
Protocatechuic acid	40	33
Vanillic acid	75	81
Gallic acid	22	22
Salicylic acid	7	12
Gentisic acid	97	89
p-Coumaric acid	75	67
Caffeic acid	1	-
Ferulic acid	76	-
Sinapic acid	87	7
Trans-resveratrol	270	170
Myricetin	5	1
Cyanidin-3-O-glucoside	90	90
Petunidin-3-O-glucoside	1	1
Malvidin-3-O-glucoside	43	47

**Table 2:** Predicted oral toxicity, nuclear receptor interactions, stress response pathways, molecular initiating events, and CYP-mediated metabolism of *trans*-resveratrol.

Classification	Target	Shorthand	Prediction	Probability
Organ toxicity	Hepatotoxicity	dili	Inactive	0.74
Organ toxicity	Neurotoxicity	neuro	Inactive	0.77
Organ toxicity	Nephrotoxicity	nephro	Active	0.59
Organ toxicity	Respiratory toxicity	respi	Inactive	0.57
Organ toxicity	Cardiotoxicity	cardio	Active	0.51
Toxicity endpoints	Carcinogenicity	carcino	Inactive	0.71
Toxicity endpoints	Immunotoxicity	immuno	Inactive	0.86
Toxicity endpoints	Mutagenicity	mutagen	Inactive	0.92
Toxicity endpoints	Cytotoxicity	cyto	Inactive	0.98
Toxicity endpoints	BBB-barrier	bbb	Inactive	0.55
Toxicity endpoints	Ecotoxicity	eco	Inactive	0.55
Toxicity endpoints	Clinical toxicity	clinical	Inactive	0.60
Toxicity endpoints	Nutritional toxicity	nutri	Inactive	0.89
Nuclear receptor	Aryl hydrocarbon Receptor (AhR)	nr_ahr	Inactive	0.63
Nuclear receptor	Androgen Receptor (AR)	nr_ar	Active	1.0
Nuclear receptor	Androgen Receptor Ligand Binding Domain (AR-LBD)	nr_ar_lbd	Inactive	0.99
Nuclear receptor	Aromatase	nr_aromatase	Inactive	0.85
Nuclear receptor	Estrogen Receptor Alpha (ER)	nr_er	Active	1.0
Nuclear receptor	Estrogen Receptor Ligand Binding Domain (ER-LBD)	nr_er_lbd	Active	1.0
Nuclear receptor	PPAR-Gamma	nr_ppar_gamma	Inactive	0.97
Stress response	Nrf2/ARE	sr_are	Inactive	0.93
Stress response	HSE	sr_hse	Inactive	0.93
Stress response	MMP	sr_mmp	Active	1.0
Stress response	p53	sr_p53	Inactive	0.53
Stress response	ATAD5	sr_atad5	Active	1.0
Molecular Initiating Events	THRα	mie_thr_alpha	Inactive	0.90
Molecular Initiating Events	THRβ	mie_thr_beta	Inactive	0.78
Molecular Initiating Events	TTR	mie_ttr	Inactive	0.97
Molecular Initiating Events	RYR	mie_ryr	Inactive	0.98
Molecular Initiating Events	GABAR	mie_gabar	Inactive	0.96
Molecular Initiating Events	NMDAR	mie_nmdar	Inactive	0.92
Molecular Initiating Events	AMPAR	mie_ampar	Inactive	0.97
Molecular Initiating Events	KAR	mie_kar	Inactive	0.99
Molecular Initiating Events	AChE	mie_ache	Inactive	0.74
Molecular Initiating Events	CAR	mie_car	Inactive	0.98
Molecular Initiating Events	PXR	mie_pxr	Inactive	0.92
Molecular Initiating Events	NADHOX	mie_nadhox	Inactive	0.97
Molecular Initiating Events	VGSC	mie_vgsc	Inactive	0.95
Molecular Initiating Events	NIS	mie_nis	Inactive	0.98
Metabolism	CYP1A2	CYP1A2	Active	0.92
Metabolism	CYP2C19	CYP2C19	Inactive	0.71
Metabolism	CYP2C9	CYP2C9	Active	0.76
Metabolism	CYP2D6	CYP2D6	Inactive	0.80
Metabolism	CYP3A4	CYP3A4	Active	1.0
Metabolism	CYP2E1	CYP2E1	Inactive	0.99

**Table 3:** Details about possible toxicity targets.

	Toxicity Target	Avg Pharmacophore Fit	Avg Similarity Known Ligands
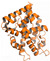	Androgen Receptor	1.27%	78.37%
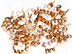	Prostaglandin G/H Synthase 1	30.82%	82.45%

**Table 4:** Impact of AQE and EtOHE of *A. boeticus* on urinary electrolyte excretion at the end of a 15-day course of treatment.

Groups	Saluretic Index			Rapport Na+/K+
	Sodium	Potassium	Chloride	
Control	1	1	1	0.67
Furosemide	1.12	1.38	1.6	0.54
EtOHE	1.02	1.12	0.93	0.60
AQE	1.08	1.3	1.24	0.55

Saluretic index = treated mmol/L/control mmol/L.

**Table 5:** Molecular characteristics of trans-resveratrol.

Property	Value
Molecular weight	228.24 g/mol
Number of hydrogen bond acceptors	3
Number of hydrogen bond donors	3
Number of atoms	17
Number of bonds	18
Number of rotatable bonds	2
Molecular refractivity	67.88
Topological polar surface area (TPSA)	60.69 Å^2^
Octanol/water partition coefficient (logP)	2.97

## Data Availability

The original contributions presented in this study are included in the article. Further inquiries can be directed to the corresponding author.
